# Round-the-Clock Adsorption–Degradation of Tetracycline Hydrochloride by Ag/Ni-TiO_2_

**DOI:** 10.3390/ma17122930

**Published:** 2024-06-14

**Authors:** Siyu Ma, Yiying Qin, Kongyuan Sun, Jahangeer Ahmed, Wei Tian, Zhaoxia Ma

**Affiliations:** 1 College of Chemistry & Environment, Southwest Minzu University, Chengdu 610225, China; masiyu1220email@163.com (S.M.); qinyiying040220@163.com (Y.Q.); kongyuansun@163.com (K.S.); 2 Department of Chemistry, College of Science, King Saud University, Riyadh 11451, Saudi Arabia; jahmed@ksu.edu.sa; 3 School of Physical Science and Technology, Soochow University, Suzhou 215006, China; wtian@suda.edu.cn

**Keywords:** adsorption, persistent photocatalytic degradation, photo-generated holes, Ag nanoparticles, TiO_2_

## Abstract

The synergy of adsorption and photocatalysis is a good method to remove organic pollutants in wastewater. In recent decades, persistent photocatalysis has gained considerable interest for its ability to sustain the catalytic degradation of organic pollutants in the dark. Herein, we report three different TiO_2_ nanomaterials to remove tetracycline hydrochloride (TCH) in solution. We found that the removal ability of TiO_2_, Ni-TiO_2_, and Ag/Ni-TiO_2_ is 8.8 mg/g, 13.9 mg/g and 23.4 mg/g, respectively, when the initial concentration of TCH is 50 mg/L. Chemical adsorption could be the rate-determining step in the TCH adsorption process. Moreover, Ag nanoparticles dispersed on Ni doped TiO_2_ surface act as traps to capture photo-generated electrons upon illumination with indoor light. The holes in Ag/Ni-TiO_2_ serve as critical oxidative species in TCH degradation under dark conditions. This work provides new insights into the design of persistent photocatalysts that can be activated by weak illumination and degrade organic pollutants in wastewater after sunset.

## 1. Introduction

Tetracycline hydrochloride (TCH) is a typical antibiotic and has long been used as a broad-spectrum antibacterial agent in the livestock, poultry, and human medical industries [1,2]. It is obtained from a common hydronaphthacene core comprising four fused rings and is difficult to degrade due to its stable chemical structure [3,4]. TCH excreted into the aqueous environment through human and animal urine, causing harm to the ecological environment and human health, has raised significant concerns [5,6]. Therefore, an efficient method is urgently needed to remove TCH residues from the environment.

Adsorption is considered as an appropriate and feasible method to remove TCH from the aqueous environment due to its economic feasibility and convenience [2,7]. Zhang et al. prepared a biochar–ceramsite composite and obtained a 30.72 mg/g adsorption capacity of TCH [7]. Kong et al. found that an MOF-525(Co) metal organic framework showed a 469.5 mg/g adsorption capacity of TCH at 298 K [6]. However, TCH enriched with adsorption materials must still be degraded. Photocatalysis is a highly efficacious way to solve this problem because of its high efficiency, deep oxidation capability, good sustainability, cost effectiveness, etc. [8]. Photogenerated reactive oxidative species (h^+^, ·O_2_^−^, ·OH, H_2_O_2_, etc.) could degrade organic pollutants and avoid secondary pollution [9]. Recently, the combination of adsorption and photocatalysis has been extensively investigated for the removal and degradation of organic pollutants in water, leveraging their complementary advantages [10,11,12]. S-defected MoS_2_ coupling with S,N-codoped porous biochar prepared by the Zheng group showed great adsorption capacity and a 97.99% removal efficiency of TCH within 120 min [12]. Lu et al. prepared a TiO_2_/(Bi_2_O_3_/Bi_2_O_2.33_) heterostructure which showed an excellent adsorption and removal efficiency of TCH [13]. Although the above-reported materials can remove TCH by synergistic adsorption–photocatalysis, a continuous supply of light is essential for the degradation of TCH. Once the irradiation stops, the photocatalyst will immediately become inactive [14].

Persistent photocatalysis is urgently needed to be developed for the application of TCH degradation during day and night. Persistent photocatalysis is mainly constructed by heterojunction systems, which are composed of a charge storage material and a photosensitive material for charge injection [14,15,16,17]. The charge storage material can store the excess photogenerated energy (h^+^ or e^−^) from the photosensitive material with light excitation and then release the stored energy without light [9]. Wang et al. prepared a multifunctional catalyst of T/NC/MoS_2_@Ag NFs which removed 97.2% tetracycline under weak irradiation by released electrons [18]. Fu et al. found that the electron storage capacity of Ti_3_C_2_/TiO_2_/Ag was up to 0.125 μmol/g, which resulted in an improved dark-reaction activity to degrade organic pollutants [15].

TiO_2_ is vigorously applied as a photosensitive material to photocatalysis owning to its excellent stability and low cost. Nonetheless, the development of TiO_2_ is hindered by its wide band gap of 3.2 eV and poor utilization of visible light [19]. Doping with metals (Ni, Cr, Fe, Co, etc.) into the TiO_2_ lattice would actively modify the TiO_2_ physical properties because of the creation of an impurity energy level [20,21,22]. Au, Pt, and Ag nanoparticles have capacitive properties that can store electrons produced in the light-reaction stage [4,17,22,23]. Moreover, a Schottky junction, formed at the metal/semiconductor interface, is a typical heterojunction [24]. It can induce the formation of surface sub-band-gap states, significantly affect the charge separation process, redistribute interfacial charges, and modulate the chemisorption of reaction intermediates [25,26].

Herein, we synthesize a new Ag/Ni-TiO_2_ composite for the efficient persistent photocatalysis of TCH in the absence of in situ irradiation light sources. Ni-doped TiO_2_, which could be activated by visible light, was selected as the photosensitive material. Ag nanoparticles were used as the trap center to store photogenerated charge carriers upon illumination. We found that chemical adsorption limited the removal efficiency of TCH during the adsorption process. Ag/Ni-TiO_2_ could be activated by weak indoor light to generate photoinduced holes as critical oxidative species in TCH degradation under dark.

## 2. Materials and Methods

### 2.1. Preparation of Materials

TiO_2_ was prepared using a hydrothermal method according to the literature [27,28]. Initially, 0.01 mol titanium sulfate (Sinopharm Chemical Reagent Co., Shanghai, China) was added to 60 mL of deionized water and stirred for 20 min. Subsequently, the mixture was transferred into a 100 mL Teflon-lined stainless-steel autoclave and sealed. After heating at 180 °C for 12 h, the precursor was separated by filtration and washed with H_2_O, and then dried at 60 °C in a vacuum oven for 12 h.

For the synthesis of Ni-TiO_2_, 0.01 mol titanium sulfate and 0.0025 mol nickel nitrate hexahydrate (Chron Chemicals, Chengdu, China) were simultaneously added to 60 mL of deionized water, and the subsequent treatment process was the same as that for the preparation of TiO_2_ mentioned above.

**Figure 1:**
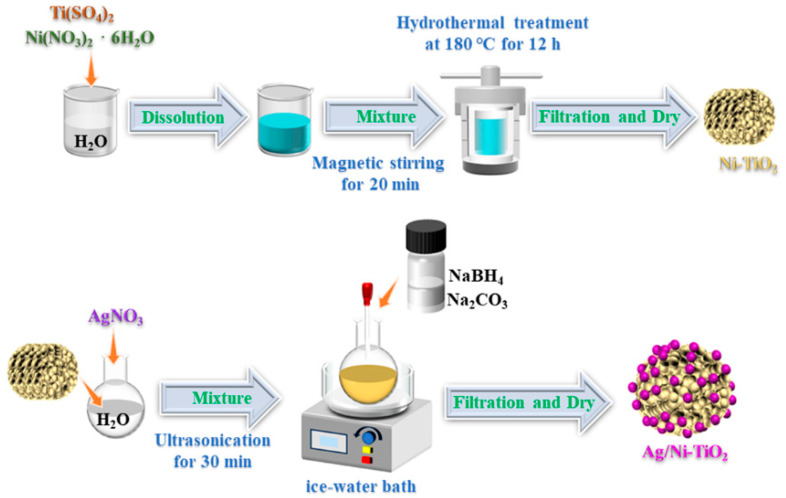
Schematic diagram of the synthesis steps of Ag/Ni-TiO_2_.

For the synthesis of Ag/Ni-TiO_2_, 100 mg Ni-TiO_2_ and 625 μL AgNO_3_ solution (0.1 mol/L) (Aladdion Biochemical Technology Co., Shanghai, China) were simultaneously added to 20 mL of deionized water, followed by ultrasonication for 30 min to form mixture A. Meanwhile, 0.047 g NaBH_4_ (Chron Chemicals, Chengdu, China) and 0.066 g Na_2_CO_3_ (Sinopharm Chemical Reagent Co., Shanghai, China) were simultaneously added to 6 mL of deionized water and stirred for 20 min to form mixture B. Then, mixture B was slowly added into mixture A in an ice-water bath under stirring. Finally, the precursor was separated by filtration and washed with H_2_O, and then dried at 60 °C in a vacuum oven for 12 h. The synthetic route is shown in Figure 1.

### 2.2. Characterization

X-ray diffraction (XRD) patterns were recorded on a Smartlab X-ray diffractometer (Rigaku, Tokyo, Japan) with Cu Kα radiation (λ = 0.15405 nm). The determination of specific surface area and pore volumes involved the utilization of N_2_ adsorption and desorption isotherms, which were conducted using a Micromeritics ASAP 2460 instrument (Micromeritics, Atlanta, GA, USA). The morphology of the samples was characterized using scanning electron microscopy (SEM, Hitachi S-4800) (Hitachi, Tokyo, Japan). X-ray photoelectron spectroscopy (XPS) was performed on a Thermo-Scientific K-Alpha (Thermo Fisher Scientific, Waltham, MA, USA). The content of Ni in Ni-TiO_2_ was determined using inductively coupled plasma-atomic emission spectrometry (ICP-AES, Agilent 720ES) (Agilent, Santa Clara, CA, USA). Fourier-transform infrared spectroscopy (FT-IR) was performed using an Agilent Cary 660 (Agilent, Santa Clara, CA, USA).

### 2.3. Adsorption and Degradation Experiment

#### 2.3.1. Adsorption–Degradation Performance of TCH

A total of 50 mg of each sample was added to a 50 mL TCH solution with different concentrations (10 mg/L, 30 mg/L, and 50 mg/L), and the adsorption experiments were performed in a crystal vessel (100 mL) at 293 K with continuous stirring. Samples were gathered at regular intervals and the concentration of TCH was assessed. This experiment was carried out in the dark.

#### 2.3.2. Adsorption–Degradation Cycles of TCH over Ag/Ni-TiO_2_

A total of 50 mg of Ag/Ni-TiO_2_ was added to a 10 mg/L aqueous TCH solution (50 mL), followed by stirring under dark for 150 min at 293 K. Then, the Ag/Ni-TiO_2_ powders were collected by high-speed centrifugalizing and vacuum drying at 60 °C, and then added to the TCH solution again. 

#### 2.3.3. Adsorption–Degradation Cycles of TCH over Illuminated Materials

A total of 50 mg of Ag/Ni-TiO_2_ (or Ni-TiO_2_) was added to a 10 mg/L aqueous TCH solution (50 mL), followed by stirring in the dark for 150 min at 293 K. Then, the Ag/Ni-TiO_2_ (or Ni-TiO_2_) powders were collected by high-speed centrifugalizing and vacuum drying at 60 °C. They were then illuminated using a 350 W Xe lamp (or Xe lamp with a wavelength exceeding 420 nm) for 2 h and then added to the TCH solution again.

### 2.4. Determination of TCH

The concentration of TCH was measured using a UV-Vis spectrometer at 358 nm [6]. The removal efficiency and adsorption capacity (*q_t_*) for TCH were calculated according to Equations (1) and (2):

(1) $$\mathrm{Removal}\;\mathrm{efficiency}=\frac{\left(C_{0}-C_{t}\right)}{C_{0}}\times 100\%$$

(2) $$q_{t}=\frac{V(C_{0}-C_{t})}{m}$$

where the initial concentration is denoted as *C*_0_, and the concentration at time *t* for TCH is denoted as *C_t_*. *V* represents the initial volume of the TCH solution (50 mL), and *m* denotes the dosage of the samples (50 mg).

## 3. Results and Discussion

**Figure 2:**
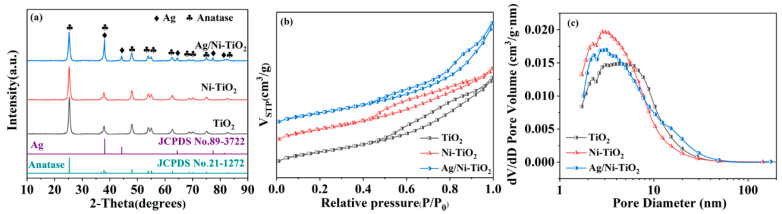
XRD (**a**), N_2_ adsorption–desorption isotherms (**b**), and pore size distribution (**c**) of TiO_2_, Ni-TiO_2_, and Ag/Ni-TiO_2_.

The crystal phases of TiO_2_, Ni-TiO_2_, and Ag/Ni-TiO_2_ are shown in Figure 2a. TiO_2_ and Ni-TiO_2_ were characterized by the anatase phase (JPCDS No. 21-1272) [29]. The doping of Ni did not alter the crystal phase structure of TiO_2_. The other five peaks centered at 38.1°, 44.3°, 64.4°, 77.4°, and 81.5° were identified as the (111), (200), (220), (311), and (222) planes corresponding to Ag (JPCDS No. 89-3722) over the Ag/Ni-TiO_2_ sample [30]. The results suggested that Ag nanoparticles dispersed on the surface of Ni-TiO_2_. 

N_2_ adsorption and desorption isotherms were conducted to analyze the porosity and specific surface area of samples. As shown in Figure 2b, all samples exhibited type Ⅳ isotherm which signifies the mesoporous nature of the samples [19]. The specific surface area of Ni-TiO_2_ was 128.34 m^2^/g, exceeding that of pure TiO_2_ at 118.77 m^2^/g. And the specific surface area of Ag/Ni-TiO_2_ was 113.90 m^2^/g, which indicates that pores on Ni-TiO_2_ were partially filled or blocked by Ag nanoparticles. The average pore diameters of TiO_2_, Ni-TiO_2_, and Ag/Ni-TiO_2_ were 7.06, 5.74, and 8.42 nm (Figure 2c), respectively, suggesting that all samples exhibited a mesoporous structure.

**Figure 3:**
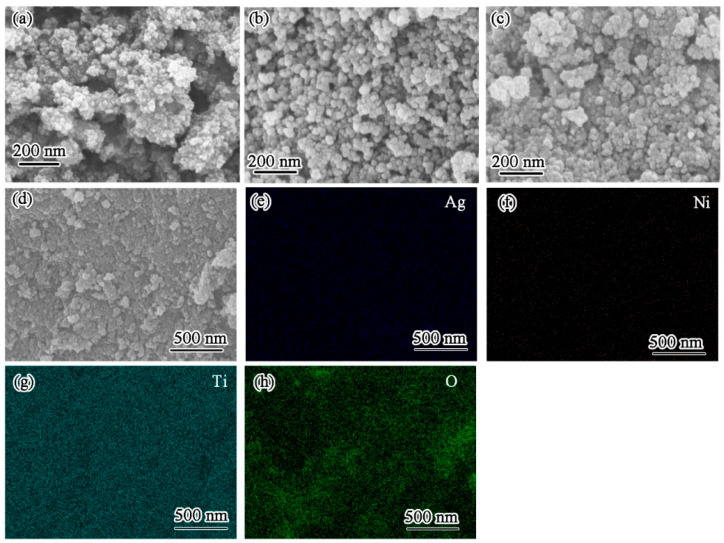
SEM images of TiO_2_ (**a**), Ni-TiO_2_ (**b**), and Ag/Ni-TiO_2_ (**c**); (**d**) SEM image of Ag/Ni-TiO_2_ at low magnification. (**e**) EDS Ag mapping of the region shown in (**d**), (**f**) EDS Ni mapping of the region shown in (**d**), (**g**) EDS Ti mapping of the region shown in (**d**), (**h**) EDS O mapping of the region shown in (**d**).

Figure 3a–d show the SEM images of TiO_2_, Ni-TiO_2_, and Ag/Ni-TiO_2_, respectively. It is evident that the agglomeration of nanoparticles occurred on the TiO_2_ sample, as shown in Figure 3a. The doping of Ni promoted the dispersion of nanoparticles in Figure 3b. The loading of Ag nanoparticles did not significantly affect the microstructure in Figure 3c,d. To verify the composition of Ag/Ni-TiO_2_, we carried out energy dispersive spectroscopic (EDS) mapping, as shown in Figure 3e–h. Chemical mapping affirms the presence of Ag, Ti, and O. According to the ICP-MS results, the content of Ni is 0.075 wt.% in Ni-TiO_2_, which results in a weak Ni mapping signal.

**Figure 4:**
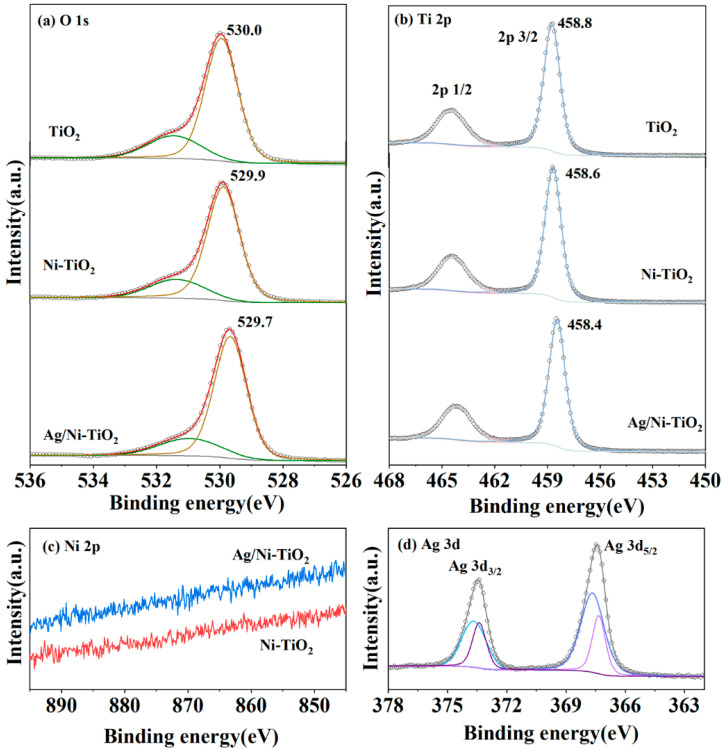
XPS spectra of TiO_2_, Ni-TiO_2_, and Ag/Ni-TiO_2_. (**a**) O 1s, (**b**) Ti 2p, (**c**) Ni 2p, and (**d**) Ag 3d.

The XPS spectra of all samples are shown in Figure 4. The wide survey XPS spectra of TiO_2_, Ni-TiO_2_, and Ag/Ni-TiO_2_ in Figure S1 indicate the presence of C, O, Ti, and Ag elements. O 1s spectra (Figure 4a) were deconvoluted into two peaks at 531.5–531.1 and 530.0–529.7 eV, assigned to adsorbed oxygen and lattice oxygen, respectively [31]. For Ti 2p, the splitting peaks at 464.4–464.2 and 458.8–454.4 eV, respectively, correspond to Ti 2p_1/2_ and Ti 2p_3/2_ of Ti^4+^ [11]. The Ni 2p signal is almost not detected over Ni-TiO_2_ and Ag/Ni-TiO_2_ in Figure 4c. However, for Ni-TiO_2_, the Ti 2p_3/2_ and lattice oxygen peaks displayed a negative shift of 0.2 eV and 0.1 eV, respectively, compared with pure TiO_2_, which was attributed to the perturbation of the doped Ni atoms in the crystal structure of TiO_2_ [32]. 

The Ag 3d XPS spectrum of Ag/Ni-TiO_2_ exhibits distinct peaks at 373.4 eV and 367.4 eV, corresponding to the Ag 3d_3/2_ and Ag 3d_5/2_ states, respectively. The binding gap between the peaks is 6 eV, which aligns with the Ag^0^ state [33]. Additionally, it should be mentioned that there is a negative shift of approximately −0.8 eV in the binding energy from 374.2 to 373.4 eV in comparison with pristine Ag. This could be attributed to the intimate contact established between Ag nanoparticles and Ni-TiO_2_, resulting in the formation of a Schottky junction, with some electrons being transferred across the barrier from Ni-TiO_2_ and trapped in Ag particles [34,35,36]. For Ag/Ni-TiO_2_, the Ti 2p_3/2_ and lattice oxygen peaks displayed a negative shift of 0.2 eV and 0.2 eV, respectively, compared with Ni-TiO_2_. Such shifts in the binding energy could be due to the adjustment of the Fermi level in the composite Ag/Ni-TiO_2_ [31,37].

**Figure 5:**
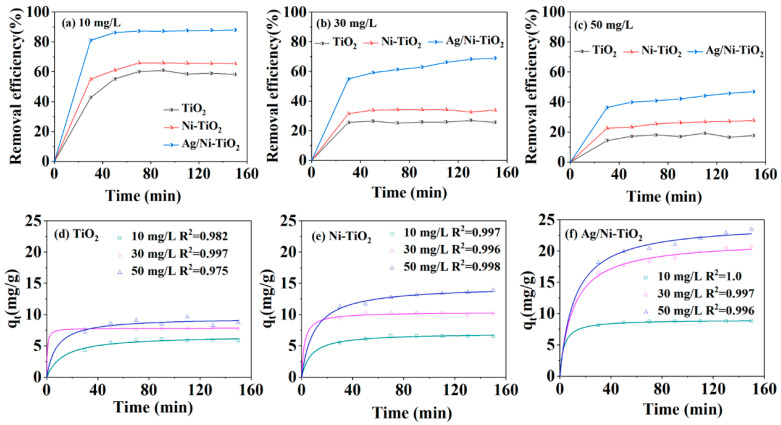
Removal efficiency of TCH over TiO_2_, Ni-TiO_2_, and Ag/Ni-TiO_2_ for the initial concentration of 10 mg/L (**a**), 30 mg/L (**b**), and 50 mg/L (**c**) in the dark. Adsorption capacity of TCH and nonlinear regressions of the PSOK model over TiO_2_ (**d**), Ni-TiO_2_ (**e**), and Ag/Ni-TiO_2_ (**f**).

Figure 5 displays the adsorption–degradation experiments of TCH over the samples under dark conditions. The removal efficiency of TCH by TiO_2_, Ni-TiO_2_, and Ag/Ni-TiO_2_ was 42.77%, 55.11%, and 81.03% at 30 min, and then reached 58.93%, 65.59%, and 87.89% at 150 min when the initial concentration of TCH was 10 mg/L, as shown in Figure 5a. It is evident that the removal efficiency decreased when the initial concentration of TCH increased to 30 and 50 mg/L over all samples, as shown in Figure 5b,c, and Ag/Ni-TiO_2_ performed optimum adsorption–degradation. 

Further, the adsorption capacity (*q_t_*) of TCH over the three samples was calculated and is displayed in Figure 5d–f. The fast adsorption occurring within the initial 30 min could be attributed to the ample adsorption sites on the surface of the samples [6]. As shown in Figure 5d, *q_t_* reached 5.9, 8.1, and 8.8 mg/g at 150 min when the concentration of TCH was 10, 30, and 50 mg/L, respectively, which indicates that the adsorption equilibrium was reached when TCH concentration was 30 mg/L on TiO_2_. However, *q_t_* kept increasing over Ni-TiO_2_ and Ag/Ni-TiO_2_ when the concentration of TCH increased, and reached 13.9 and 23.4 mg/g, respectively, at 150 min when the concertation of TCH was 50 mg/L (Figure 5e,f). The above results indicate that the doping of Ni atoms and Ag nanoparticles strengthens the removal performance of TiO_2_. 

To study the adsorption kinetics during TCH removal, the removal efficiency over the Ag/Ni-TiO_2_ composite between 0 and 40 min was tested. The adsorption kinetics were well matched with the pseudo-second-order kinetic model (PSOK) due to higher correlation coefficient (R^2^) values, as shown in Figure S2. Therefore, the PSOK model is used to describe the adsorption kinetics over three samples. As shown in Figure 5d–f, the R^2^ values for all samples are approaching 1.0, which suggests that the chemical adsorption could be the rate-determining step in the TCH adsorption process over all samples [38,39,40]. Moreover, according to the BET results in Figure 2b, Ag/Ni-TiO_2_ has the smallest specific surface area, which further demonstrates that physical adsorption does not limit the removal of TCH.

**Figure 6:**
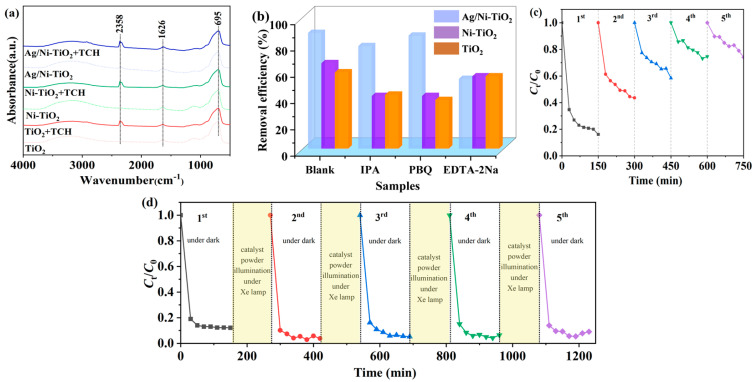
(**a**) FT–IR spectrum results of the samples before and after TCH adsorption. (**b**) Free radical capture experiments. (**c**) Adsorption–degradation cycles of TCH over Ag/Ni-TiO_2_ under dark conditions. (**d**) Adsorption–degradation cycles of TCH under dark conditions; the cycled Ag/Ni-TiO_2_ was irradiated using a Xe lamp for 2 h.

As shown in Figure 6a, the composition of fresh and TCH-adsorbed samples was analyzed using FT-IR tests. The peak at 695 cm^−1^ corresponds to the stretching vibration of Ti-O-Ti [40]. The adsorption peaks at 1626 and 3200 cm^−1^ are attributed to -OH and H_2_O, respectively [22]. It can be seen that adsorbed CO_2_ at 2358 cm^−1^ was detected over TCH-adsorbed samples [41], which indicates that the adsorbed TCH can undergo dark degradation, resulting in the production of CO_2_.

In order to further clarify the above proposal, free radical capture experiments in the dark were carried out. Isopropyl alcohol (IPA), 1,4-Benzoquinone (PBQ), and ethylenediaminetetraacetic acid sodium (EDTA-2Na) were used as scavengers for hydroxyl radicals, superoxide radicals, and holes, respectively [11,42]. As shown in Figure 6b, over the TiO_2_ sample, after adding IPA, PBQ, and EDTA-2Na, the removal efficiency of TCH decreased from 58% to 41%, 37%, and 55%, respectively. The results indicate that hydroxyl radicals and superoxide radicals, which could be formed from the active oxygen in the solution [43], are the main active species to degrade TCH to CO_2_ over the TiO_2_ surface. Over the Ni-TiO_2_ sample, after adding IPA, PBQ, and EDTA-2Na, the removal efficiency of TCH decreased from 65% to 40%, 40%, and 55%, respectively, which suggests that the doping of Ni atoms could promote the generation of holes to degrade TCH. Over the Ag/Ni-TiO_2_ sample, after adding IPA, PBQ, and EDTA-2Na, the removal efficiency of TCH decreased from 88% to 78%, 86%, and 53%, respectively, which suggests that holes generated on the surface of Ag/Ni-TiO_2_ are the main active species leading to the removal of TCH in the dark. It is worth noting that all samples were only irradiated by an indoor light during the preparation process.

To better demonstrate the removal ability of Ag/Ni-TiO_2_, an adsorption–degradation cycle of TCH was conducted for five times, as exhibited in Figure 6c. The removal efficiency of TCH reached 84%, 56%, 42%, 25%, and 25% in the first, second, third, fourth, and fifth process, respectively. The results suggest that the adsorption capacity reached saturation after the third iteration. The removal efficiency of 25% in the fourth and fifth processes could be caused by the degradation of TCH by holes over Ag/Ni-TiO_2_. The XRD pattern, SEM image, and EDS mapping of cycled-Ag/Ni-TiO_2_ are shown in Figures S3–S5. It can be seen that the crystal phase and micromorphology of the composite remained unchanged. The results indicate that the Ag/Ni-TiO_2_ composite had excellent stability during the adsorption–degradation cycle of TCH.

Further, the cycled Ag/Ni-TiO_2_ was irradiated with a Xe lamp for 2 h and then placed into the TCH solution again for the degradation application in the dark. As shown in Figure 6d, the removal efficiency of TCH remained at about 95% during five cycle processes in the dark. When the cycled Ag/Ni-TiO_2_ was irradiated using a Xe lamp with a wavelength exceeding 420 nm for 2 h, as shown in Figure S6, the removal efficiency of TCH degraded to 85% in the fourth cycle, which could be attributed to the loss of Ag nanoparticles on the surface. Furthermore, an adsorption–degradation cycle of TCH over Ni-TiO_2_ was conducted, as shown in Figures S7 and S8. The removal efficiency of TCH increased to 90% during the cycles when the cycled Ni-TiO_2_ powder was irradiated with a Xe lamp for 2 h. When the cycled Ni-TiO_2_ powder was irradiated using a Xe lamp with a wavelength exceeding 420 nm for 2 h, the removal efficiency of TCH remained at about 70% during the cycles. The above results indicated that Ni-TiO_2_ and Ag/Ni-TiO_2_ could be activated by pre-illumination with visible light to store enough charge for the effective and persistent degradation of TCH under dark conditions. UV light illumination could enhance the stored charge on the surface of the composite. The higher performance of Ag/Ni-TiO_2_ could be attributed to the formation of a Schottky junction at the interface according to the XPS results. This junction facilitates the transfer of excited electrons to the Ag nanoparticles because of the silver’s higher work function, effectively boosting the efficiency of charge separation and leaving energetic positive charges in Ni-TiO_2_, which essentially function as holes for the oxidation of TCH in the dark [22,44,45].

## 4. Conclusions

In summary, we have reported the excellent ability of a Ag/Ni-TiO_2_ composite for the persistent photodegradation of TCH with good recycling capability. The removal capacity of TCH reached 23.4 mg/g in the presence of an indoor light-irradiated Ag/Ni-TiO_2_ composite. We found that adsorption and degradation simultaneously occurred during TCH removal and chemical adsorption could be the rate-determining step in the TCH adsorption process. The loaded Ag nanoparticles act as trapping sites to capture photo-generated electrons upon illumination. The holes in Ag/Ni-TiO_2_ served as critical oxidative species in TCH degradation in the dark. This work provides new insights into the design of persistent photocatalysts that can be activated by weak visible light and degrade organic pollutants after sunset.

## Data Availability

The original contributions presented in the study are included in the article and Supplementary Materials, further inquiries can be directed to the corresponding author.

## Supplementary Materials

The following supporting information can be downloaded at: https://www.mdpi.com/article/10.3390/ma17122930/s1, Figure S1: Survey XPS spectra of TiO_2_, Ni-TiO_2_, and Ag/Ni-TiO_2_; Figure S2: Nonlinear regressions of the pseudo-first-order kinetic model (PFOK) (a) and pseudo-second-order kinetic model (PSOK) (b) over Ag/Ni-TiO_2_ between 0 and 40 min; Figure S3: XRD of Ag/Ni-TiO_2_ and cycled-Ag/Ni-TiO_2_; Figure S4: SEM of cycled-Ag/Ni-TiO_2_; Figure S5: (a) SEM image of cycled-Ag/Ni-TiO_2_ at low magnification, (b) EDS Ag mapping of the region shown in (a), (c) EDS Ni mapping of the region shown in (a), (d) EDS Ti mapping of the region shown in (a), (e) EDS O mapping of the region shown in (a); Figure S6: Adsorption–degradation cycles of TCH under dark condition, the cycled-Ag/Ni-TiO_2_ was irradiated using a Xe lamp with a wavelength exceeding 420 nm for 2 h; Figure S7: Adsorption–degradation cycles of TCH under dark condition, the cycled-Ni-TiO_2_ was irradiated using a Xe lamp for 2 h; Figure S8: Adsorption–degradation cycles of TCH under dark condition, the cycled-Ni-TiO_2_ was irradiated using a Xe lamp with a wavelength exceeding 420 nm for 2 h.
