# Supplementary material for: Round-the-Clock Adsorption–Degradation of Tetracycline Hydrochloride by Ag/Ni-TiO2
Source: Materials (Basel). 2024 Jun 14;17(12):2930. doi: 10.3390/ma17122930 (PMC11205127; doi:10.3390/ma17122930)
Supplement: Supplementary file 1 [file materials-17-02930-s001.zip › materials-3020865-supplementary.pdf]

Round-the-Clock Adsorption–Degradation of Tetracycline Hydrochloride by Ag/Ni-TiO_2

Siyu Ma [1], Yiying Qin [1], Kongyuan Sun [1], Jahangeer Ahmed [2], Wei Tian [3] and Zhaoxia Ma [1,*]

[1] College of Chemistry & Environment, Southwest Minzu University, Chengdu 610225, China
[2] Department of Chemistry, College of Science, King Saud University, Riyadh 11451, Saudi Arabia
[3] School of Physical Science and Technology, Soochow University, Suzhou 215006, China
* Correspondence: zxmamse@163.com

S1. Adsorption kinetics

First-order (Eq. (S1)) and second-order (Eq. (S2)) models were employed to evaluate the adsorption kinetic behaviors of TCH.

First order:

$$q_{\mathrm{t}} = q_{\mathrm{e}}(1 - \mathrm{e}^{-k_{\mathrm{f}}t}) \quad \text{(S1)}$$

Second order:

$$q_{\mathrm{t}} = q_e^2\mathrm{k_s t}/(1 + q_{\mathrm{e}}\mathrm{k_s t}) \quad \text{(S2)}$$

where k_f (min^{-1}) and k_s(min^{-1}) are the rate constants of the first order and second order, and q_e is the amount of adsorbate adsorbed at equilibrium.

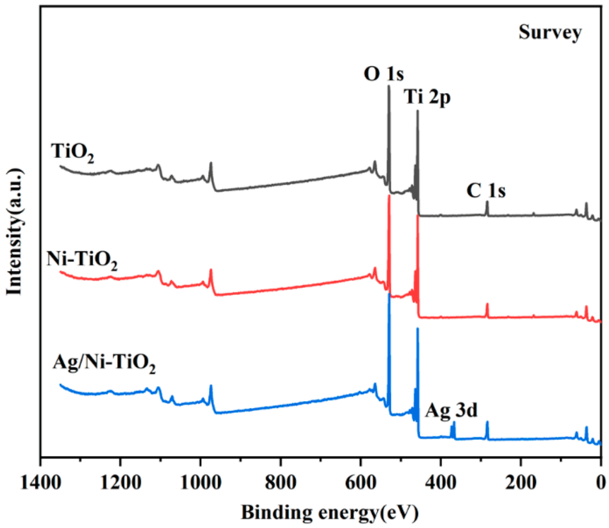

Figure S1. Survey XPS spectra of TiO_2, Ni-TiO_2, and Ag/Ni-TiO_2.

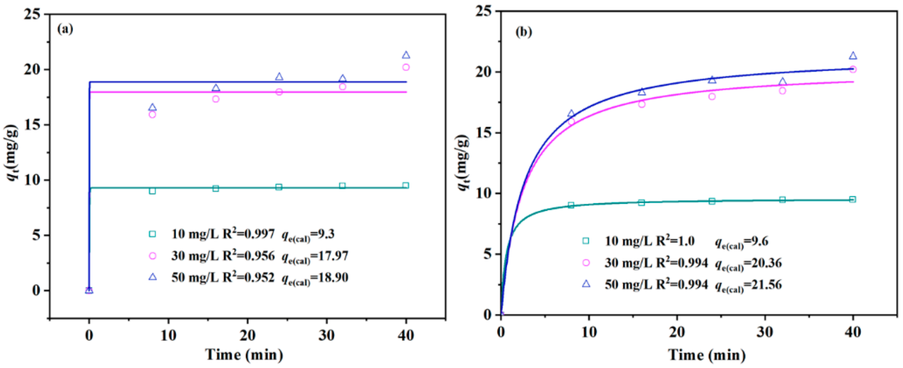

Figure S2. Nonlinear regressions of the pseudo-first-order kinetic model (PFOK) (a) and pseudo-second-order kinetic model (PSOK) (b) over Ag/Ni-TiO_2 between 0 and 40 min.

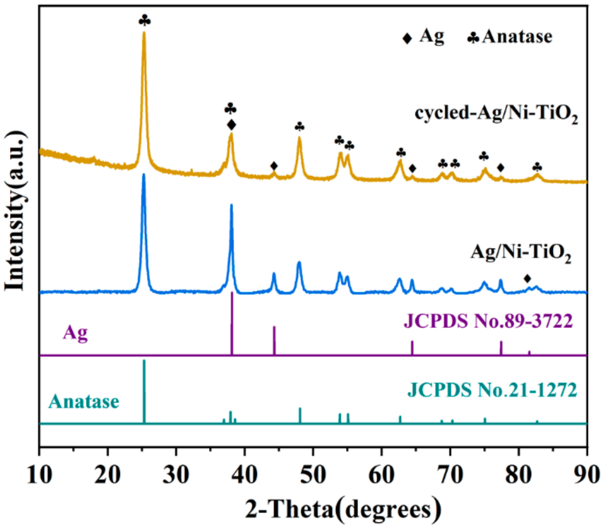

Figure S3. XRD of Ag/Ni-TiO_2 and cycled Ag/Ni-TiO_2.

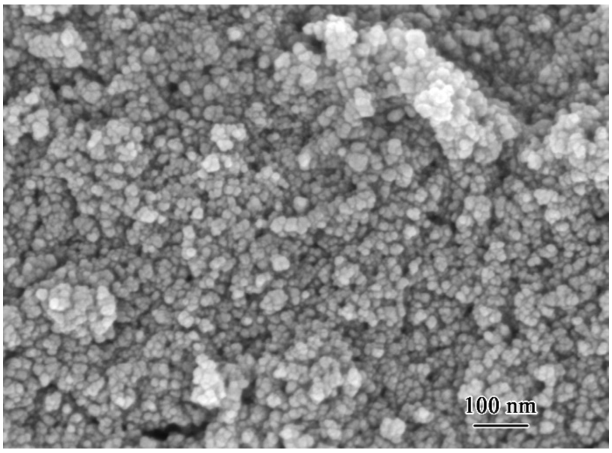

Figure S4. SEM of cycled Ag/Ni-TiO_2.

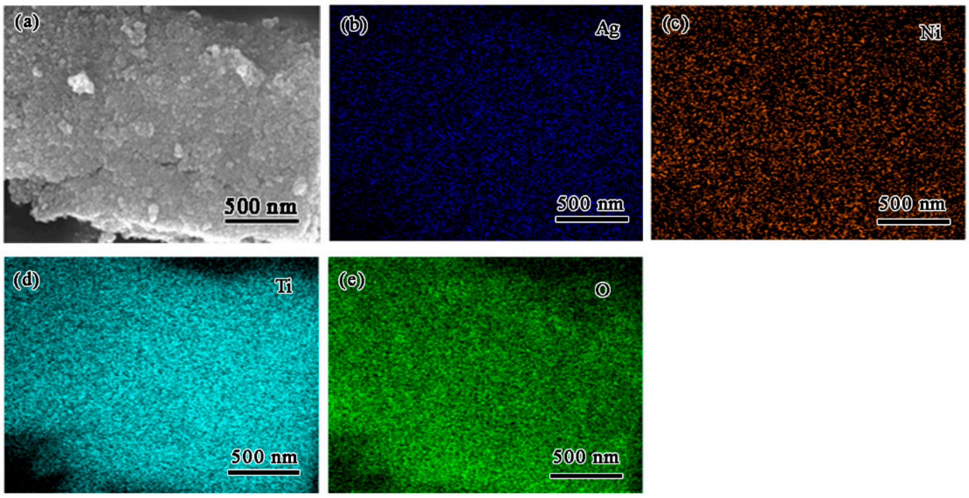

Figure S5. (a) SEM image of cycled Ag/Ni-TiO_2 at low magnification. (b) EDS Ag mapping of the region shown in (a), (c) EDS Ni mapping of the region shown in (a), (d) EDS Ti mapping of the region shown in (a), and (e) EDS O mapping of the region shown in (a).

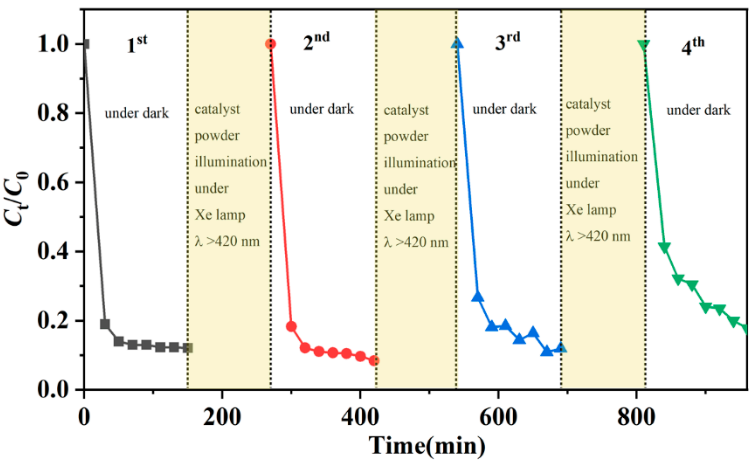

Figure S6. Adsorption–degradation cycles of TCH under dark conditions; the cycled Ag/Ni-TiO_2 was irradiated using a Xe lamp with a wavelength exceeding 420 nm for 2 hours.

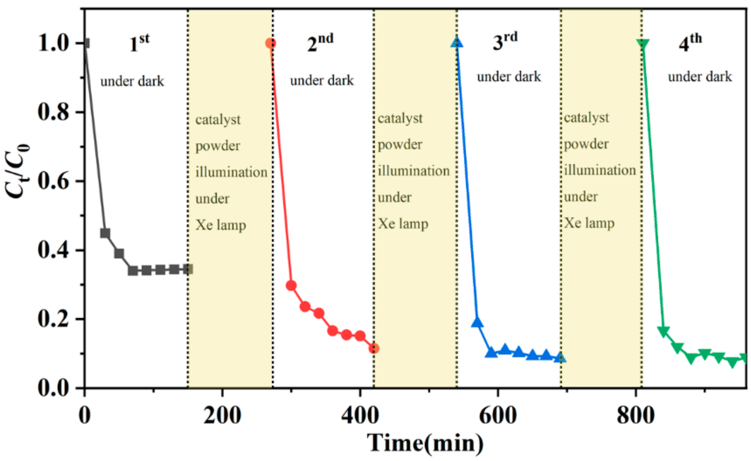

Figure S7. Adsorption–degradation cycles of TCH under dark conditions; the cycled Ni-TiO_2 was irradiated using a Xe lamp for 2 hours.

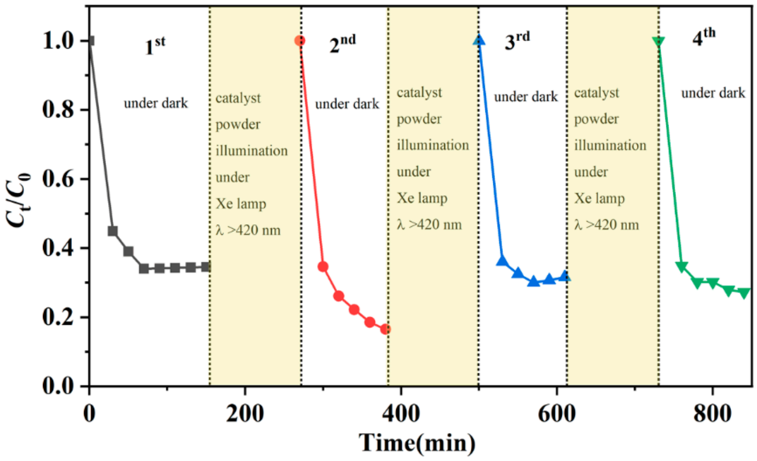

Figure S8. Adsorption–degradation cycles of TCH under dark conditions; the cycled Ni-TiO_2 was irradiated using a Xe lamp with a wavelength exceeding 420 nm for 2 hours.
